# Trends in incidence and survival of childhood cancers in Khon Kaen, Thailand (2000–2019): a population-based Khon Kaen Cancer Registry study

**DOI:** 10.1186/s12889-024-18742-0

**Published:** 2024-05-07

**Authors:** Chanaporn Pinsuwan, Chalongpon Santong, Su-on Chainansamit, Patcharee Komvilaisak, Prapassara Sirikarn, Surachai Phimha, Kunanya Suwannaying

**Affiliations:** 1 Doctor of Public Health Program, Faculty of Public Health, Khon Kaen University, 123 Mittraphap road, Muang Khon Kaen, Khon Kaen, 40002 Thailand; 2 Khon Kaen Cancer Registry, Cancer unit, Srinagarind Hospital, Faculty of Medicine, Khon Kaen University, 123 Mittraphap road, Muang Khon Kaen, Khon Kaen, 40002 Thailand; 3 Department of Pediatrics, Khon Kaen Hospital, 54 Sri Chant road, Muang Khon Kaen, Khon Kaen, 40000 Thailand; 4 Department of Pediatrics, Srinagarind Hospital, Faculty of Medicine, Khon Kaen University, 123 Mittraphap road, Muang Khon Kaen, Khon Kaen, 40002 Thailand; 5 Department of Epidemiology and Biostatistics, Faculty of Public Health, Khon Kaen University, 123 Mittraphap road, Muang Khon Kaen, Khon Kaen, 40002 Thailand; 6 Department of Public Health Administration, Health Promotion, and Nutrition, Faculty of Public Health, Khon Kaen University, 123 Mittraphap road, Muang Khon Kaen, Khon Kaen, 40002 Thailand

**Keywords:** Trends in incidence, Relative survival, Childhood cancers, Population-based

## Abstract

**Background:**

In Thailand, the national health care system and nationwide standard treatment protocols have evolved over time, potentially influencing the trends in the incidence and survival rates of childhood cancers. However, further investigations are required to comprehensively study these trends in Khon Kaen, Thailand.

**Methods:**

Childhood cancer patients aged 0–14 years (*n* = 541) who were diagnosed with one of the five most common cancers between 2000 and 2019 from the population-based Khon Kaen Cancer Registry were enrolled. Descriptive statistics were used to analyse the demographic data, which are presented as numbers, percentages, means, and standard deviations. The trends in incidence between 2000 and 2019, including age-standardized incidence rates (ASRs) and annual percent changes (APCs), were analysed using the Joinpoint regression model. Survival analysis was performed for 5-year relative survival rates (RSRs) according to the Pohar Perme estimator and Kaplan–Meier survival curves.

**Results:**

The ASRs of the overall top 5 childhood cancer groups were 67.96 and 106.12 per million person-years in 2000 and 2019, respectively. Overall, the APC significantly increased by 2.37% each year for both sexes. The overall 5-year RSRs were 60.5% for both sexes, 58.2% for males, and 63.9% for females. The highest 5-year RSR was for germ cell tumours (84.3%), whereas the lowest 5-year RSR was for neuroblastoma (29.1%).

**Conclusions:**

The incidence and survival rates of childhood cancers in Khon Kaen, Thailand, varied according to sex. The incidence trends increased over time, meanwhile, the relative survival rates rose to satisfactory levels and were comparable to those of other nations with similar financial status. The implementation of national health policies and adherence to national treatment guidelines have improved cancer diagnosis and treatment outcomes.

**Supplementary Information:**

The online version contains supplementary material available at 10.1186/s12889-024-18742-0.

## Background

Although childhood cancers are uncommon, they are considered to be one of the most common causes of mortality among children aged 0–14 years in Thailand [1]. The mortality rate by childhood cancer type ranged from 0.88 to 5.08 deaths per 100,000 population in 2019, emphasizing the considerable impact of this health issue [1]. During the period of 1990–2000, the 5-year overall survival rate for childhood cancers in Thailand was only 39.4% [2]. Several factors impede the effective treatment of childhood cancer, thereby obstructing the path to achieving desired health outcomes. These include limited health literacy, constrained resources affecting accessibility and diagnostic accuracy, inadequate supportive care, the stigma associated with cancer, and broader social hardships. Together, these challenges contribute to difficulties in accessing health care services and can ultimately lead to patients abandoning therapy [3].

To enhance treatment outcomes, the National Health Security Office (NHSO) implemented the Universal Coverage Scheme (UCS) in 2001, achieving full coverage in 2002, encompassing a majority of Thai people, including children [4, 5]. In 2006, the NHSO initiated and supported a nationwide disease management program for childhood leukaemia and lymphoma, subsequently expanding and updating it to include protocols for all types of childhood cancer. These protocols have been implemented nationwide since 2016. As a result, accessibility to health care facilities and advanced treatments for childhood cancer patients substantially improved [6, 7].

Globally, including in Thailand, the incidence of childhood cancer has increased, but mortality has decreased [8]. However, the trends in the incidence and survival of childhood cancer in Khon Kaen, Thailand, a city located in an area of high poverty and health care inequality, require comprehensive study [9], particularly following changes in health policy. This study aimed to evaluate (1) the trend in the incidence and (2) the relative survival rates of childhood cancers in Khon Kaen from 2000 to 2019. Insights from this study could enhance the understanding of the impact of national health policies and treatment guidelines, leading to the development of strategies to improve the health care system in areas with limited resources.

## Methods

### Data quality

The population-based Khon Kaen cancer registry covers 26 districts in Khon Kaen Province, Thailand. Childhood cancer data were collected from 23 community hospitals, 3 private hospitals, Khon Kaen Hospital, and Srinagarind Hospital. The data were reviewed by the Khon Kaen Cancer Unit. The quality of the Khon Kaen cancer registry data consisted of four components according to the National Cancer Institute Thailand: comparability, completeness, validity, and timeliness. The quality of the data was assessed by 4% death certificates only (DCO), and 90% morphological verification (MV) was within the acceptable range. Furthermore, the percentage of MV has increased, while DCO and clinical-based diagnosis have decreased [10].

### Study population

The study participants were children under 15 years of age in Khon Kaen, Thailand, diagnosed with leukaemia, lymphoma, central nervous system (CNS) neoplasms, neuroblastoma, or germ cell tumours between January 1st, 2000, and December 31st, 2019. The exclusion criteria included incomplete data for the year of last follow-up and last vital status, household registrations in provinces other than Khon Kaen, and previous cancer treatment at hospitals outside Khon Kaen. The population denominator for age-standardized rates (ASRs) was calculated using the population at risk of children aged 0–14 years in Khon Kaen Province between 2000 and 2019; these patients were classified every 5 years based on age and sex.

### Data collection

The data were obtained from the population-based Khon Kaen cancer registry. Newly diagnosed childhood cancer patients aged 0–14 years were identified using the International Classification of Diseases for Oncology, third edition (ICD-O-3) and then categorized into the International Classification of Childhood Cancer, third edition (ICCC-3), which includes the top five childhood cancers. The data collected included age at cancer diagnosis, sex, current status, residential area, and health insurance. The accrual period was from January 1st, 2000, to December 31st, 2019, and continued survival follow-up until December 31st, 2021. Since 2000, the Khon Kaen Cancer Registry database was reliable and completed as well as the essential improvements in health policy, treatment protocol, and healthcare coverage in Thailand.

### Statistical analysis

Descriptive statistics were used to analyse the demographic data, which are presented as numbers and percentages for categorical data and as the means and standard deviations (SDs) for continuous data. For trend in incidence analysis, ASRs between 2000 and 2019 were analysed using the Joinpoint regression model and are represented in trend graphs. The Walter and Elwood test was applied to investigate the cyclical trend [11]. The annual percent change (APC) was used to provide an overview of trend change between 2000 and 2019, if there was no cyclicity. Trend analysis was conducted using the Joinpoint Regression Program version 5.0.2 [12].

Survival analysis was performed for 5-year relative survival rates, median survival times, and Kaplan–Meier survival curves. The data analyses were conducted in Stata version 15 [13].

## Results

### Demographic data

This study included 541 of 578 patients who were diagnosed with leukaemia, lymphoma, CNS neoplasms, neuroblastoma, or germ cell tumours between 2000 and 2019. Thirty-seven patients (6.4%) were excluded because of missing data for the year of last follow-up and last vital status. The most common cancers were leukaemia (53.8%), followed by CNS neoplasms (19.0%), lymphoma (10.7%), germ cell tumours (9.2%) and neuroblastoma (7.2%). The majority of these patients were male (58.2%). The mean age at diagnosis was 6.48 years (SD = 4.50), and the median age was 6 years (range 0–14). Approximately 40% were aged 0 to 4 years. Approximately 51% of the patients were diagnosed between 2010 and 2019. The majority of patients (95.2%) had coverage under the Universal Coverage Scheme (UCS) for health insurance. Almost 69% of the participants resided in areas located more than 79.9 km away from the city centre (Table 1).

**Table 1:** Patient demographic data (*n* = 541)

Variable	Number	Percentage
Sex		
Male	315	58.23
Female	226	41.77
Age at diagnosis (years)		
0–4	220	40.67
5–9	156	28.83
10–14	165	30.50
Mean (SD)	6.48 (4.50)	
Median (Min: Max)	6 (0: 14)	
Period of diagnosis (10 years)		
2000–2009	263	48.61
2010–2019	278	51.39
Childhood cancer group		
Leukaemia	291	53.79
CNS neoplasms	103	19.04
Lymphoma	58	10.72
Germ cell tumours	50	9.24
Neuroblastoma	39	7.21
Health insurance (*n* = 497)		
Universal Coverage Scheme (UCS)	473	95.17
Civil Servant Medical Benefit Scheme (CSBMS)	24	4.83
Residential area (distance from the city centre)		
Short and intermediate distance (< 20 km to 79.9 km)	372	68.76
Long distance (≥ 80 km)	169	31.24

*Abbreviations: km* Kilometre, *Min* Minimum, *Max* Maximum, *SD* Standard deviation

### The incidence rates and trends of childhood cancer

**Fig. 1:**
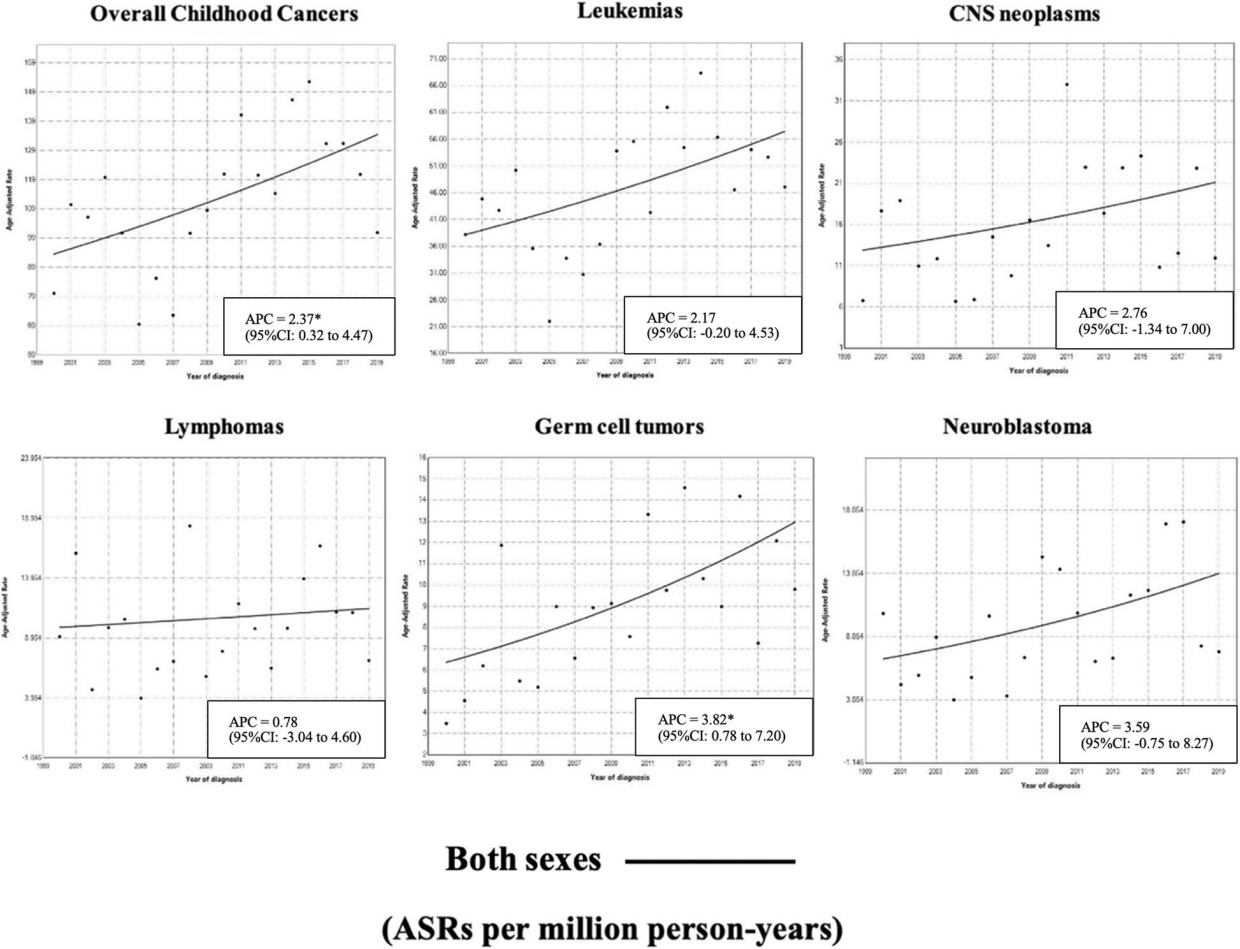
Trends in incidence of childhood cancers in Khon Kaen between 2000 and 2019

The ASR of the overall five most common childhood cancers was 68.0 per million person-years in 2000 and increased to 106.1 per million person-years in 2019. In 2000, the overall ASRs of leukaemia, lymphoma, CNS neoplasms, neuroblastoma, and germ cell tumours were 38.2, 9.9, 13.2, 7.1, and 6.4 per million person-years, respectively. By 2019, the ASRs had increased to 57.5, 11.4, 22.1, 13.9, and 13.0 per million person-years, respectively. Therefore, the incidence trends were demonstrated in the model of 0-joinpoint due to the obvious point and overview explanation provided by the single line (Fig. 1). In the leukaemia subgroup, acute lymphoid leukaemia (ALL) and acute myeloid leukaemia (AML) had ASRs of 29.2 and 9.3 per million person-years, respectively, in 2000, before increasing to 36.3 and 12.4 per million person-years, respectively, in 2019. By the lymphomas subgroup, the ASRs of Hodgkin lymphoma (HL) and non-Hodgkin lymphoma (NHL) were 4.2 and 5.7 per million person-years, respectively, in 2000, before increasing to 6.0 and 11.2 per million person-years, respectively, in 2019. The ASRs by sex of the overall five most common childhood cancers from 2000 to 2019 were 82.2 to 115.3 per million person-years in males and 54.8 to 100.9 per million person-years in females.

The incidence of the five most common childhood cancers increased significantly by 2.37% annually between 2000 and 2019 (95% CI: 0.32% to 4.47%). The APCs in the incidence of leukaemia, lymphoma, CNS neoplasms, and neuroblastoma not statistically significant increased annually for both sexes by 2.17% (95% CI: -0.20% to 4.53%), 0.78% (95% CI: -3.04% to 4.60%), 2.76% (95% CI: -1.34% to 7.00%), and 3.59% (95% CI: -0.75% to 8.27%), respectively, while the incidence of germ cell tumours increased significantly for both sexes by 3.82% (95% CI: 0.78% to 7.20). For the leukaemia subgroup, APCs for ALL and AML not statistically significant climbed by 1.14% (95% CI: -1.36% to 3.53%) and 1.55% (95% CI: -2.69% to 5.76%), respectively. Among the lymphomas subgroup, the APCs of HL and NHL rose statistically significantly for both sexes by 1.95% (95%CI: 0.57 to 3.29%) and 3.63% (95%CI: 0.30% to 7.17%), respectively. When stratified by sex, females had a greater increase in the incidence rate of all cancers except lymphoma. The incidence of neuroblastoma significantly increased by 4.36% (95% CI: 1.37% to 7.58%) among females (Table 2).

**Table 2:** Annual percent change (APC) in the incidence of childhood cancer by ICCC group and sex from 2000 to 2019

ICCC group	Overall	Sex	
		**Male**	**Female**
Overall top 5 childhood cancer groups	2.37^a^ (0.32 to 4.47)	1.80 (-1.19 to 4.80)	3.26 (-0.36 to 7.07)
I. Leukaemia	2.17 (-0.20 to 4.53)	1.90 (-1.26 to 5.07)	2.50 (-0.88 to 5.87)
II. Lymphoma	0.78 (-3.04 to 4.60)	1.56 (-1.72 to 4.79)	1.41 (-1.58 to 4.32)
III. Central nervous system neoplasms	2.76 (-1.34 to 7.00)	2.43 (-0.31 to 5.18)	3.71 (-0.63 to 8.24)
IV. Neuroblastoma	3.59 (-0.75 to 8.27)	2.35 (-1.80 to 6.48)	4.36^a^ (1.37 to 7.58)
X. Germ cell tumours	3.82^a^ (0.78 to 7.20)	2.61^a^ (0.28 to 4.95)	3.82^a^ (1.37 to 6.49)

*Abbreviation: ICCC* International Classification of Childhood Cancer

^a^Statistically significant at the level of 0.05

### Survival

**Fig. 2:**
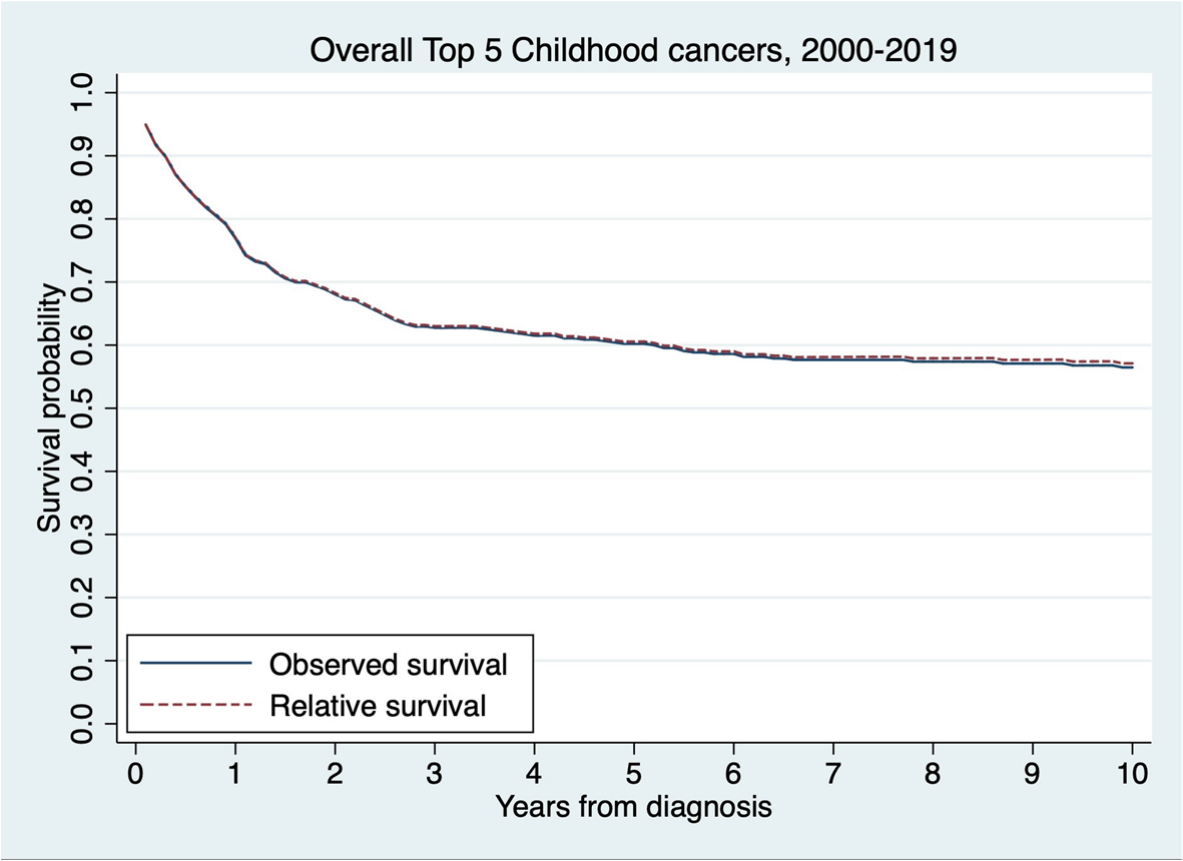
Observed and relative survival of the overall top 5 childhood cancers in Khon Kaen, Thailand, between 2000 and 2019

The relative survival rates (RSRs) of patients with overall five most common childhood cancers between 2000 and 2019 at 1, 3, 5, and 10 years were 77.0% (95% CI: 73.2% to 80.4%), 63.0% (95%: 58.7% to 67.0%), 60.5% (96% CI: 56.2% to 64.6%), and 57.1% (95% CI: 52.5% to 61.4%), respectively (Fig. 2). The overall 5-year RSRs were 58.2% (95% CI: 52.4% to 63.5%) in males and 63.9% (95% CI: 57.0% to 69.9%) in females, with significant differences by sex (*p*-value for log-rank test = 0.045).

**Fig. 3:**
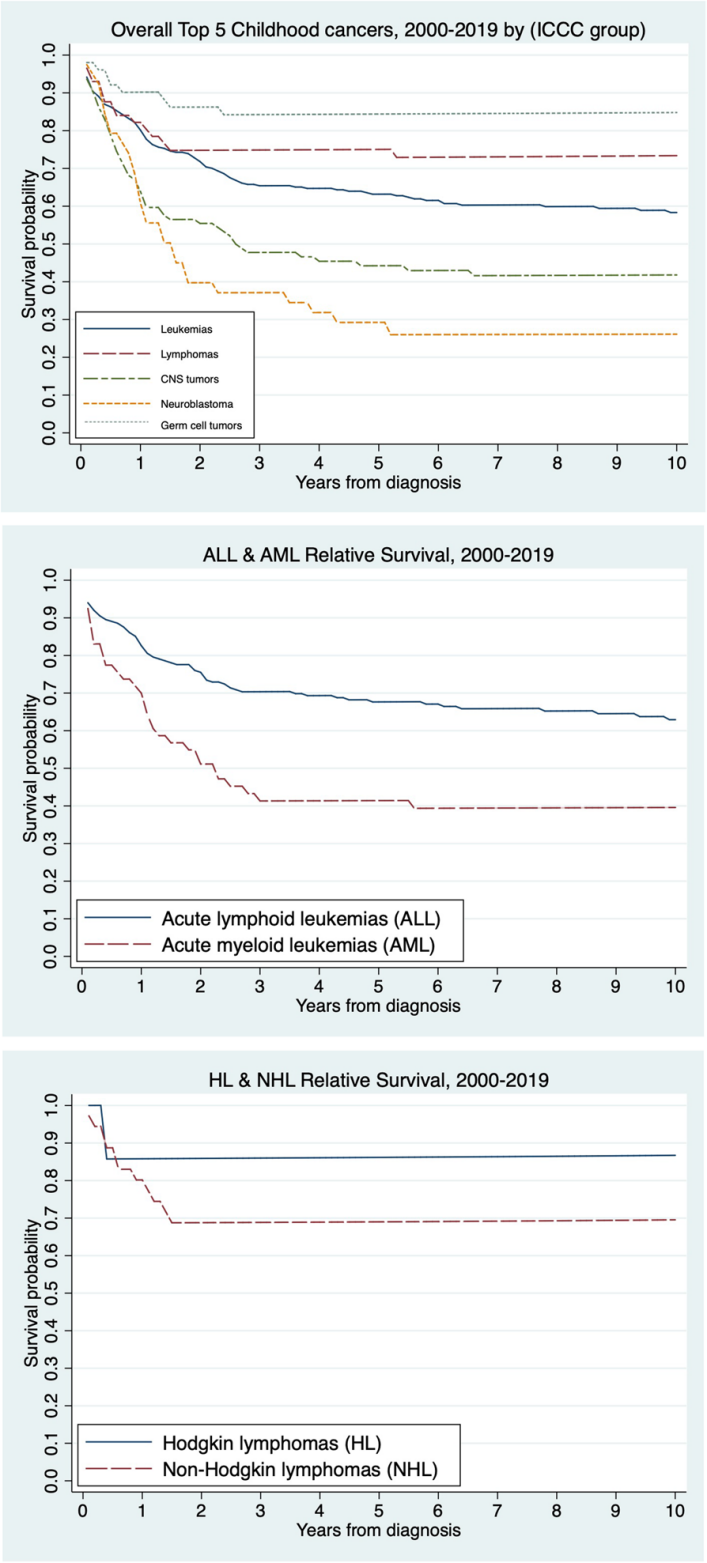
**a** Relative survival of the overall top 5 childhood cancers in Khon Kaen, Thailand, between 2000 and 2019 by ICCC group. **b** Relative survival of leukemias in Khon Kaen, Thailand, between 2000 and 2019 by subgroups of acute lymphoid leukemias (ALL) and acute myeloid leukemias (AML). **c** Relative survival of lymphomas in Khon Kaen, Thailand, between 2000 and 2019 by subgroups of Hodgkin lymphomas (HL) and non-Hodgkin lymphomas (NHL)

A comparison of the relative survival of the five most common childhood cancers in Khon Kaen by ICCC group between 2000 and 2019 revealed that the good prognosis were germ cell tumours (84.3%; 95% CI: 70.6% to 92.0%), lymphomas (75.1%; 95% CI: 61.3% to 84.6%), and leukaemia (63.1%; 95% CI: 57.2% to 68.4%). The poor prognosis was neuroblastoma (29.1%; 95% CI: 15.9% to 43.8%), followed by CNS neoplasms (44.4%; 95% CI: 34.1% to 54.1%) (Fig. 3a) (Table 3). The median survival times of CNS neoplasms and neuroblastoma were 2.59 and 1.53 years, respectively. The median follow-up times were 9.43 years for germ cell tumours, 7.27 years for lymphomas, 5.55 years for leukaemia, 2.48 years for CNS neoplasms, and 1.36 years for neuroblastoma. The percentages of censored were 82.0% for germ cell tumours, 72.4% for lymphomas, 58.8% for leukaemia, 45.6% for CNS neoplasms, and 28.2% for neuroblastoma. The greater details of childhood cancer patients in the last status at the end of the follow-up period were provided in Supplemental Table 1. Among the leukaemia subgroups, ALL patients had a higher 5-year RSR (67.5%; 95% CI: 60.4% to 73.6%) than AML patients (41.6%; 95% CI: 28.4% to 54.3%) (Fig. 3b). For lymphomas, the 5-year RSR was 86.2% (32.3% to 98.1%) for HL and 69.2% (51.1% to 81.7%) for NHL (Fig. 3c).

**Table 3:** Overall 5-year relative survival rates by ICCC group

ICCC group	Overall 5-year RS (%) **(95% CI)**
Overall top 5 childhood cancers	60.5 (56.2 to 64.6)
I. Leukaemia	63.1 (57.2 to 68.4)
II. Lymphoma	75.1 (61.3 to 84.6)
III. Central nervous system neoplasms	44.4 (34.1 to 54.1)
IV. Neuroblastoma	29.1 (15.9 to 43.8)
X. Germ cell tumours	84.3 (70.6 to 92.0)

*Abbreviations: CI* Confidence interval, *ICCC* International Classification of Childhood Cancer, *RS* Relative survival

## Discussion

This population-based study revealed an upward trend in the incidence of the five most common childhood cancers overall in Khon Kaen between 2000 and 2019, with an annual increase of 2.37% each year from 2000 to 2019. This trend is consistent with patterns observed in the Thailand database [2, 6] and in many countries worldwide [8, 14–16]. The success of universal health care coverage implementation in reducing socioeconomic inequalities in health care access, particularly in rural Thailand, is demonstrated by this trend [17]. Furthermore, advances in diagnostic techniques and cancer registration have contributed to the increasing incidence of cancer. On the other hand, the ASRs of overall and each cancer declined from 2015 to 2019. The incidence trend changes in recent years might also be attributed to adjustments in diagnostic, coding, or registration procedures. As an instance, several additional cases of pilocytic astrocytoma could have been recorded as astrocytoma NOS or glioma NOS (both with the malignant behaviour code) [15, 18]. Additionally, the Thai economy fluctuated during the era, and the socioeconomic status of the patient’s family who lived outside of the capital caused many children lost to cancer detection [19].

In our study, leukaemia was the most common cancer diagnosed, and the APC in the incidence of leukaemia increased (2.2% each year) for both sexes. This finding aligns with that from a study from Korea, which observed a significant increase in leukaemia cases between 1999 and 2011, with APCs of 1.9% for males and 2.1% for females [14]. A similar trend in leukaemia incidence from 1990 to 2011, with an APC of 1.5%, was also reported in a multicancer registry analysis in Thailand [2]. The high ASR and APC of leukaemia in Khon Kaen may indicate improvements in case diagnoses, particularly in the classification of leukaemia cases in the registry, health care accessibility, and the introduction of technologies such as cytochemistry and molecular diagnostics for leukaemia diagnosis. Additionally, environmental factors such as air pollution may contribute to the increased incidence of childhood leukaemia [20]. In Khon Kaen, a rapidly urbanizing and industrializing area in Northeast Thailand, increased air pollution has resulted from swift industrial expansion and the emergence of various transport systems. High levels of particulate matter (PM2.5)-bound heavy metals, including manganese, aluminium, lead, copper, cadmium, iron, and zinc, have been found in residential and industrial areas in Khon Kaen, posing potential carcinogenic risks to children [21]. A previous study reported that paternal occupational exposure to pesticides, animals, and organic dust is associated with an increased risk of acute myeloid leukaemia (AML) [22]. However, the direct association between environmental factor exposure and the incidence of childhood leukaemia requires further investigation.

In addition, we observed upward trends in all cancers, with significant increases in germ cell tumours for both sexes and neuroblastomas for females (*p* < 0.05). Similar trends were found in nationwide studies in Thailand [2], Estonia [16] and Austria [23]. These trends may be attributed to enhanced diagnostic methods, leading to earlier investigations of slow-growing tumours. The introduction of noninvasive techniques, such as tumour marker detection, has been beneficial in the diagnostic process. Additionally, the widespread availability and easy accessibility of computed tomography (CT) and magnetic resonance imaging (MRI) have continued to play important roles in the detection and characterization of tumours [24, 25].

Our study revealed greater survival rates for most cancers, except for neuroblastoma, than did a multicancer registry analysis in Thailand [2]. The 5-year RSRs in our study were 67.5% for ALL, 41.6% for acute myeloid leukaemia (AML), 86.2% for Hodgkin lymphoma (HL), 69.2% for non-Hodgkin lymphoma (NHL), 44.4% for CNS neoplasms and 84.3% for germ cell tumours. These rates are higher than those of a previous study (2001–2011), which reported lower RSRs: 52.5% for ALL, 26.5% for AML, 60.6% for HL, 49.1% for NHL, 31.6% for CNS neoplasms and 63.4% for germ cell tumours. Regarding neuroblastoma, both our study and nationwide data (29.1% versus 27.6%) revealed similar poor prognoses, largely because most patients presented with high-risk features. The standard treatment for high-risk neuroblastoma involves multiagent chemotherapy, surgery, autologous haematopoietic stem cell transplantation (HSCT), radiation, and immunotherapy [7]. Unfortunately, not all neuroblastoma patients have access to HSCT, and immunotherapy is currently unavailable in our country.

In the context of Khon Kaen, despite high poverty levels and inequality of health care, the implementation of universal health care coverage and nationwide guidelines has substantially improved patient access to health care. These upward trends in the incidence and survival rates are indicative of the success of Thailand’s national health policy and treatment standards [26–28]. Nonetheless, survival rates in this study and the entire of Thailand are still lower than those in developed countries [15, 29, 30], possibly due to disparities in accessibility to diagnostic and health care services, as well as other resource constraints [31, 32]. Furthermore, advanced medical technologies for cancer treatment are unavailable in this region such as hematopoietic stem cell transplantation (HSCT) and immunotherapy [33]. On the other hand, disparities in childhood cancer infrastructure between nations with developed and developing economies contribute to variations in medical training and expertise, impacting early cancer detection [19, 32, 34].

The strength of this study is that all patient data included in this study were from the Khon Kaen Cancer Registry, which was established in 1984 and has been quality controlled for comparability, completeness, validity, and timeliness by the National Cancer Institute Thailand, with DCO and MV percentages falling within the acceptable range. However, this study has several limitations. First, data collection for childhood cancer patients is scarce, especially for early data predating the establishment of the cancer registry. Some older records of patients before the year 2000 were excluded due to incomplete data for analysis. Second, certain risk factors that could impact disease outcomes, such as sociodemographic and environmental factors, were not included in this study. To identify these risk factors and address knowledge gaps for improving childhood cancer outcomes, further research on sociodemographic and other risk factors in childhood cancers is recommended.

## Conclusion

The trends in the incidence of childhood cancers in Khon Kaen, Thailand, have increased over time. The relative survival rates have risen to a satisfactory level, which could be comparable in countries with similar economic status. Rates of incidence and survival vary depending on sex. The implementation of national health policies and adherence to national treatment guidelines have improved childhood cancer diagnosis and treatment outcomes.

### Supplementary Information

Supplementary Material 1.

## Data Availability

The data that support the findings of this study are available from the Khon Kaen Cancer Registry, but restrictions apply to the availability of these data, which were used under license for the current study and are not publicly available. However, the data are available from the authors upon reasonable request and with permission of the Khon Kaen Cancer Registry.

## Abbreviations

ALL: Acute lymphoblastic leukaemia
AML: Acute myeloid leukaemia
APC: Annual percent change
ASR: Age-standardized rate
CI: Confidence interval
CNS: Central nervous system
CSBMS: Civil Servant Medical Benefit Scheme
CT: Computed tomography
DCO: Death certificate only
HL: Hodgkin lymphoma
HSCT: Haematopoietic stem cell transplantation
ICCC-3: International Classification of Childhood Cancer, third edition
ICD-O-3: International Classification of Diseases for Oncology, third edition
KM: Kilometre
MRI: Magnetic resonance imaging
MV: Morphological verification
NHL: Non-Hodgkin lymphoma
NHSO: National Health Security Office
RSR: Relative survival rate
SD: Standard deviation
UCS: Universal Coverage Scheme
