# Supplementary material for: Trends in incidence and survival of childhood cancers in Khon Kaen, Thailand (2000–2019): a population-based Khon Kaen Cancer Registry study
Source: BMC Public Health. 2024 May 7;24:1255. doi: 10.1186/s12889-024-18742-0 (PMC11077803; doi:10.1186/s12889-024-18742-0)
Supplement: Supplementary file 1 — Supplementary Material 1. [file 12889_2024_18742_MOESM1_ESM.docx]

**Supplement Table legend:**

**Supplement Table 1** Number and percentage of childhood cancer patients in last status at

the end of the follow-up period

**Supplement Table 1** Number and percentage of childhood cancer patients in last status at

the end of the follow-up period

| **Childhood cancer group** | **Number of patients’ status (%)** | | |
| --- | --- | --- | --- |
|  | **Dead** | **Alive** | **Loss** |
| I. Leukaemia (n=291) | 120 (41.2) | 118 (40.6) | 53 (18.2) |
| I(a) Acute lymphoid leukaemia (ALL) (n=200) | 73 (36.5) | 95 (47.5) | 32 (16.0) |
| I(b) Acute myeloid leukaemia (AML) (n=53) | 33 (62.3) | 5 (9.4) | 15 (28.3) |
| II. Lymphomas (n=58) | 16 (27.6) | 32 (55.2) | 10 (17.2) |
| II(a) Hodgkin lymphoma (HL) (n=7) | 1 (14.3) | 6 (85.7) | 0 (0.0) |
| II(b) Non-Hodgkin lymphoma (NHL) (n=36) | 12 (33.3) | 15 (41.7) | 9 (25.0) |
| III. CNS neoplasms (n=103) | 56 (54.4) | 28 (27.2) | 19 (18.4) |
| IV. Neuroblastoma (n=39) | 28 (71.8) | 5 (12.8) | 6 (15.4) |
| X. Germ cell tumours (n=50) | 9 (18.0) | 37 (74.0) | 4 (8.0) |
